# Care without co‐presence: Crafting alternative modes of involvement in UK intensive care in times of COVID‐19

**DOI:** 10.1111/maq.70066

**Published:** 2026-05-25

**Authors:** Annelieke Driessen, Lisa Hinton

**Affiliations:** ^1^ Research Group Smart Health, School of Health Saxion University of Applied Sciences Deventer/Enschede Overijssel the Netherlands; ^2^ Nuffield Department of Primary Care Health Sciences University of Oxford Oxford UK

**Keywords:** COVID‐19, intensive care, modes of involvement, separation, subject positions

## Abstract

In this article we analyze how family involvement in intensive care in the United Kingdom (UK) was reconfigured through the reordering of proximity and distance during the first year of the COVID‐19 pandemic, and the effects thereof. The introduction of visiting restrictions disrupted established modes of involvement in intensive care, prompting family members, hospital staff and, when able, patients, to craft alternative modes of involvement. Drawing on narrative interviews with patients and family members, some of whom had clinical training themselves, we describe three ways in which they did so: establishing connections and communication, personalizing care at a distance, and making kin and virtual co‐presence. We conclude that the ensuing subject positions afforded involvement of some kind, but also came at a cost. Our analysis furthers the conceptual understanding of care practices beyond their reliance on proximity and distance, and highlights that their choreography shapes new possibilities as well as vulnerabilities.

## INTRODUCTION

Care is often conceptualized as dependent on physical proximity—for example, as required for ‘bodywork’ (Twigg, 2000) and touch, through which emotional closeness can be established (Kelly et al., 2017; Driessen et al., 2021)—while distance is frequently associated with ‘cold care’ (Pols & Moser, 2009). Several studies have sought to challenge these associations of warm and cold, as well as the dichotomy between distance and proximity, research on telemedicine in Spain and the Netherlands highlighting practices in which proximity is enacted through webcams and other digital devices (Aceros et al., 2014; Pols, 2012; Pols & Moser, 2009) and ethnographic work with transnational Indian families demonstrates how frequent calling serves as an informal way of enacting care and proximity despite physical distance (Ahlin, 2020, 2023). Sociological work in the context of cystic fibrosis in person care in the United Kingdom (UK) demonstrates that distance, when situated in relations of knowing, remembering, and recognizing patients, can be caring (Buse et al., 2020). Works like these highlight the need to empirically attend to how distance and proximity are variously enacted and negotiated in care practices and the effects thereof.

In this article we explore an “extreme case” of care at a distance: that of family involvement in the care for COVID‐19 patients admitted to intensive care units (ICUs) during the first months of the pandemic in the UK. In early 2020, the new viral condition COVID‐19 was highly infectious, and the mode of transmission was still unknown. The government introduced strict infection control measures in March 2020 and extended these as infection rates rose.^1^ Hospitals in turn instituted visiting restrictions,^2^ intended to contain the spread of the virus and to help the UK's National Health Service (NHS) cope with the limited availability of staff, COVID‐19 tests, and personal protective equipment (PPE). While these restrictions were introduced as a care measure,^3^ the enforced physical separation disrupted family involvement in ICUs (Buse et al., 2020).

Drawing on narrative interviews with patients and family members conducted during the first and second pandemic waves in the UK (March 2020–February 2021), we analyze how family^4^ involvement was reconfigured through shifting proximities and distance. To do so, we think with John Law's conceptualization of care as a “choreography of separation” (2010), considering how different “objects of care”—patients, family, staff, and the wider public —were differently protected through the (re)ordering of distance and proximity and what subject positions ensued. To conclude, we attend to the ambivalences that arose from the normative expectations of existing and emergent subject positions where these were incompatible. Our analysis furthers the conceptual understanding of care practices beyond their reliance on proximity and distance, and highlights that their choreography shapes possibilities as well as vulnerabilities.

## FAMILY INVOLVEMENT IN INTENSIVE CARE

ICUs are technological clinical spaces, where critically ill patients receive life‐sustaining intensive medical interventions and care. In the best of times, they are alien places for patients that evoke feelings of dependence, anxiety, uncertainty, and liminality (Egerod et al., 2015; Topçu et al., 2017). Though taking different shapes across heterogeneous settings, family involvement in ICUs is increasingly recognized as essential to ICU care, a recognition amplified by the COVID‐19 pandemic. Nursing scholars have highlighted four elements of significance regarding family participation in ICUs: the importance of physical closeness and caregiving participation; trust in care provision; the need for support in caregiving roles; and feelings of vulnerability (Blom et al., 2013). Research shows that family presence often benefits both patients and their relatives, even though family‐patient and staff‐family interactions can be complex and sometimes contentious. Families can provide emotional support (Azoulay et al., 2003; Jeitziner et al., 2022; Molter, 1994), companionship (Luttik et al., 2020), and act as advocates and translators for incapacitated patients (Curtis & White, 2008; Reeves et al., 2015, p. 235).

Resisting a universalized notion of family involvement, anthropologists have contributed situated ethnographies of care for critically ill kin. Harris Solomon's ethnography in a low‐resource public hospital in Mumbai (2021) demonstrates how family are engaged in what he calls “relational movements of breath”—making breath move between people, and machines in practices of resuscitation, intubation, and weaning—and that, in doing so, they are vital to resuscitation or ventilation practices, commonly delineated as purely clinical. In highlighting that family, if given the chance, can be more than “visitors,” Solomon's work complicates any simplistic separation of clinically trained actors from those without clinical training and invites attention to the positions of family members and others close to the hospitalized patient in ICU care.

The COVID‐19 pandemic introduced pressures on resource and clinical staff across the globe and dramatically transformed ICU environments and family involvement in care. In the UK's NHS the sudden physical distancing of family members from the ICU barred previously established modes of involvement and shifted subject positions of family members and clinical staff alike. Visiting restrictions were enforced between March 2020 and October 2022 (Boulton et al., 2022; *National Health Service England & NHS Improvement*, 2020). Having such measures in place meant that family members could no longer visit patients, bring food, presents or other items, or speak in person to clinical staff. This caused relatives significant distress (Bartoli et al., 2021; Rose et al., 2020), rendering them reliant on time‐constrained clinicians for updates. The fragmentation of family‐staff relations also impacted clinical staff emotionally (Block & Karb, 2024; Digby et al., 2021; Dowrick et al., 2021; Montgomery et al., 2021; Rodriguez, 2023). Although awareness and recognition of the importance of family members’ contributions to intensive care predated the pandemic, the sudden absence of visitors in the ICU was deeply felt by clinical staff, relatives, and patients. This absence amplified clinical staff's recognition of the value of family involvement, particularly in medical decision‐making and in providing insights into the patient's medical history, personality, and values (Driessen et al., 2020; Rodriguez, 2023). COVID‐19 ICU patients were yet differently affected: often mechanically ventilated for long periods, heavily sedated, they lacked familiar voices that could otherwise have helped to orient them, which increased the likelihood of intense delirium and hallucinations when compared to other ICU patients (Kotfis et al., 2020; Pun et al., 2021).

## CARE AND SUBJECTIVITIES

John Law explores how care was done and where it breaks down in another period marked by infectious disease: the 2001 foot‐and‐mou**t**h disease outbreak (Law 2010). He analyzes a series of photos and a commentary about the culling of animals on a farm in the south of England. Mobilizing Charis Cussins’ notion of “choreography” (1996), coined to emphasize that routines of practice are organized, Law conceptualizes the animals, the farmer, the vet himself, and the “bigger picture” as partially overlapping “objects of care”. He argues that veterinary care in the foot and mouth epidemic is best understood as a “choreography of separation” (Law 2010: 68): the farm must be kept separate from the outside world, the farmer kept away from the killing, and the children taken away from the scene entirely. In other words, bodies, technologies, architectures, texts and gestures, as well as “the subjectivities that go with them” (ibid.), must be variously kept apart. In contrast to theorizing (good) care as entailing the bringing together of particular things, Law argues that care also entails keeping them apart, and that in doing so, different subject positions are cared for.

Law's proposition that distancing is part of care practices rather than opposed to them helps to make sense of the shifting modes of family involvement in intensive care practices during the COVID‐19 pandemic. Multiple “objects of care”—patients with and without COVID‐19, hospital staff, visitors, the wider public, the economy, and perhaps even the immediate fate of the government at that time—had to be cared for. Thinking about distance and proximity as enacted through shifting choreographies of distance and proximity helps us to flesh out how different subject positions were cared for and where this broke down.

In contrast to the objects of care on the farm, the objects of care on the ICU were not straightforwardly separable and neither were the subject positions that came with them: family members could become carers for staff, staff could become kin to patients, and both could become patients themselves. This created marked ambivalences within and between subject positions that those involved were able and willing to take (Pols, 2005; Driessen, 2018, 2023). Inspired by Robert Merton's conceptualization of “ambivalence” as capturing an incompatibility of normative expectations assigned to a social position (Merton, 1976, 7), we analyze these in terms of the normative expectations to flesh out incompatibilities and social cost.

## METHODS: STUDYING EXPERIENCES OF COVID‐19 AND ICU

We draw on accounts collected in our study “Learning for improvement from patient and family members’ experiences of intensive care with Covid,” wherein we recruited a maximum variation sample,^5^ predominantly via selected NHS trusts and patient organizations. Recruiting clinicians told us they found it hard to ask patients and families who were upset or distressed about their time in the ICU (a group we suspect overlaps with those unable to be connected during the hospital stay). We therefore suspect that organizing recruitment in this manner seems to have precluded outright ‘negative’ accounts of ICU care. Hence, to include a broader range of narratives, recruitment was simultaneously done via Facebook and X (formerly Twitter), and through snowballing. The sample includes 30 people who had been admitted to intensive care with COVID‐19 in the UK from March 2020 to February 2021 (20 men and 10 women—including two who were also family to a [former] ICU COVID‐19 patient and three who worked in a clinical setting), as well as 14 family members of ICU COVID‐19 patients (13 of whom self‐identified as women and 1 as a man; two of whom were bereaved and two who (had) worked in clinical settings). The study was supported by an advisory panel, consisting of former ICU COVID‐19 patients and family members, researchers, and ICU clinicians, who reviewed the study's design, progress, and preliminary outcomes.

Annelieke (first author) conducted 40 narrative interviews between February and December 2021. Four interviews were conducted with two interviewees simultaneously, while all others were with single interviewees. 37 interviews were conducted remotely via videoconferencing platforms MS Teams and Zoom, and two were conducted in person in the participants’ homes (at a time when public health measures allowed for this), to accommodate the interviewee's preference and digital illiteracy, respectively. 39 interviews were included for analysis.

Participants provided verbal consent to participate in and video or audio‐record the interview. Each interview consisted of two parts: in the first part, interviewees were invited to tell their story, with very few interruptions by the interviewer. In the second part, Annelieke asked questions based on a topic guide, probing into admission, experiences in the ICU, discharge, and recovery in more detail. After particularly distressing interviews, Annelieke and Lisa (second author) had a debrief online.

Interview recordings were transcribed, and participants were asked to verify the transcripts and given the chance to mark excerpts for exclusion, after which they were asked for written consent. Annelieke uploaded corrected transcripts into the analysis software package, NVivo10. Annelieke and Lisa met regularly to develop the coding frame and identify key themes through a discussion of recurring themes in and surprising aspects of the interviews. Annelieke coded all interview transcripts and wrote up the analyses in the form of summaries of key themes, including “first symptoms,” “admission to the ICU,” “experiences of infection control measures,” “care at a distance” and “end‐of‐life visits,” each illustrated by a selection of quotes. Lisa reviewed the summaries alongside the corresponding coded data. Ethical approval for the UK research was obtained from the Berkshire Ethics Committee (REC reference number: 12/SC/0495).

Two online focus group discussions guided by a codesign method known as “Evidence‐Based Co‐Design” (Locock et al., 2014) with two patients and five ICU clinicians were held to explore how findings on the key themes “mitigating loneliness in the ICU” and “recovery after the ICU” could inform health care improvement initiatives. Ethics approval for the focus groups was granted by the London School of Hygiene and Tropical Medicine (LSHTM) Ethics board (Reference number: 22693).

The analyses of the twenty‐two most prominent themes comprised the module “Experiences of Intensive and COVID‐19”^6^ on the website hexi.ox.ac.uk (formerly “Healthtalk”), a web‐based resource where visitors can see and hear people's real‐life experiences of a range of illnesses and other health‐related issues. In this article we draw primarily on the data coded under the key themes “admission to the ward and ICU,” “staying in touch during the visitor ban,” “contact between partners/families and clinical staff” and “care at a distance” and the focus group discussion on “mitigating loneliness in the ICU.” Data were analyzed through a focus on how distance and proximity reconfigured family involvement, and vice versa. Combining data from patient and family interviews with focus group data in our analysis allowed us to further situate findings in ICU care practices.

## CRAFTING MODES OF INVOLVEMENT: COVID‐19 AND ICU NARRATIVES

In this section we describe three practices of crafting alternative modes of involvement in care when familiar ones were disrupted—(A) establishing connections and communication, (B) personalizing care at a distance, and (C) making kin and virtual co‐presence. We do so through the narratives of Flora, Amina, Nisha, and Suri, and supporting excerpts from other narratives. We trace how crafting alternative modes of involvement enacted new relationships and subject positions and with what effects. But before we do so, we begin by providing insights into the isolation and loneliness that gave rise to these engagements.

Emily^7^, a woman in her late forties, was admitted to the ICU in March 2020. She was immediately mechanically ventilated, which required her to be sedated. Later, she was placed on CPAP (“continuous positive airway pressure”), a machine that delivers oxygen using a set pressure to the airways following the patient's own spontaneous rhythm of breathing. In contrast to mechanical ventilation, CPAP does not necessarily require patients to be sedated, and so Emily was mostly conscious while in the ICU and witnessed what was going on around her. She described the chaos of those early days of the COVID‐19 pandemic as “half routine and half panic … like you're all in a war zone together.”

She found that staff kept their distance whenever they could and knew they were themselves afraid of contracting COVID‐19 and infecting others around them. Coupled with the physical absence of friends and family members, this meant that she spent weeks without any physical proximity to others and significantly, without skin‐to‐skin touch. Emily craved “human contact” and “would have killed for a cuddle.” PPE made the faces of staff members unrecognizable, adding to a sense of alienness and distance. Other patients similarly experienced PPE as a barrier because it made it more difficult to understand what staff said and because it increased confusion, particularly when patients came out of sedation or drifted in and out of sleep.

In more than one way, ICU COVID‐19 patients were different “objects of care” than pre‐pandemic ICU patients. Before the pandemic, it had been uncommon for ICU patients to be admitted for such long periods, let alone be awake for extended periods of time. This change increased the need and desire for emotional and physical proximity. At the same time, physical proximity was also beset with guilt and fear: patients expressed feeling an acute sense of guilt about exposing others to the virus, even after supplies of adequate PPE had stabilized.

Family members of COVID‐19 patients admitted to ICUs in March and April 2020 shared equally complex feelings. In these early weeks, contact between staff and patients’ next of kin initially occurred by happenstance, rather than by design, as initially there was little guidance from government, NHS trusts, or individual hospitals on how to overcome the sudden physical separation between staff and family members. Family members were sometimes completely in the dark regarding the whereabouts and condition of their relative in the hospital. Flora, a 48‐year‐old speech therapist, wife, and mother of two, shared a particularly striking account of her struggle for updates about her husband Theo following his admission in March 2020. On the last day before the national lockdown, Flora's and Theo's daughter called home from school to say she felt unwell. By the evening of the next day, all family members had a temperature. Flora and the children recovered within a week, but Theo became increasingly unwell. On the ninth day, he was admitted to the hospital. In the hours that followed, Flora could neither reach Theo, nor get through to the hospital. In her despair to find out where and how Theo was, Flora called various friends who worked as clinicians to ask if they knew anybody at the hospital who could look out for him. Finally, she learned that he had arrived in the hospital and had been given antibiotics and oxygen. Although she was told she would soon receive a phone call, she did not hear any clinical updates again for several days.

In the days after his admission, Theo updated Flora via calls, text messages, and photos. Soon, however, speaking on the phone became too strenuous for him. As Theo's condition worsened, he could no longer send texts, and again Flora was left without any information about his condition. Here the limits of what critically ill patients could contribute to their family members’ involvement become palpable. Flora tried to call the ward many times, but as she did not have a direct number, this meant listening to a recorded tape every time. When she finally got through, there was little consistency in who Flora spoke to; she counted twenty‐four doctors and sixty‐four nurses in total, writing down their names and job titles each time there was an update. Like many others we interviewed, Flora carried her phone with her everywhere, anxiously waiting for updates while also dreading bad news. The isolation of lockdown intensified her sense of uncertainty. Even months later, Flora found thinking back to this frantic, uncertain time difficult and upsetting.

Other family members expressed a similar intensity of emotions. Julia, whose husband Andrew was in the hospital with COVID‐19 for four months, including three months on mechanical ventilation, described feeling deeply alone. She remembered feeling an overwhelming jealousy and sense of injustice when she met a nurse at reception who could spend time with Andrew while Julia herself was actively prevented from doing so. Illustrating the ambivalence of her position, Julia said she “would not have changed it,” but this did not take away her desperation at the physical separation from her critically ill husband.

The sense of “not knowing” could persist even if family members did receive regular updates. Amina, a 38‐year‐old mother of four young children, whose husband Omar and father Ahmed had both been hospitalized in quick succession, received regular telephone updates, but she found the information fragmented and difficult to piece together. For instance, when she was told there would be no further escalation of Ahmed's treatments and that the next 24 hours on CPAP would be critical to judge how he was doing, she felt there was little context or subsequent communication about how his condition was developing. She also could not always understand where a change in treatment plan had come from—a clear break in the social and cultural expectations that doctors inform families of treatment plans and changes, such as is common in the UK's NHS. Fortunately, Ahmed began to respond positively to the CPAP ventilation, after which he could be weaned off the CPAP and transferred to the ward. Unable to see the rapid and frequent changes in his condition for herself, Amina experienced the update on his improvement not as evidence of his fluctuating condition, but rather as an inconsistency in the information she was given. Because of her physical distance from the ward, these fluctuations were not embodied for her, and she found it hard to trust clinicians’ judgments.

These stories narrate the profound impacts that visiting restrictions had on patients, family members and health care workers during the COVID‐19 pandemic, especially when somebody was dying or nearing death. Patients received care and treatment by clinical staff on the ward with several physical barriers in place, yet felt extremely lonely and isolated—illustrating that proximity was entangled at once with care, risk, and fear. Next, we turn to how patients, family members, and staff members sought to (re‐)establish family involvement in care while distanced from the ICU.

## ESTABLISHING CONNECTIONS AND COMMUNICATION

To illustrate how family members sought to regain a position from which they could provide care, we return to Flora's story. To craft an alternative mode of involvement in the care for Theo—indeed, *any* form of involvement—Flora called the hospital twice or thrice a day to get updates on his condition. She knew that the handover between the nightshift and dayshift would take place at about eight o'clock, during which staff could answer the phone. Frequently calling the hospital, Flora emphasized, quickly became for her “a ritual”; she felt that it was “the only way [she] could look after him.”

A week after Theo's admission, a doctor called Flora to say that Theo had a 50/50 chance of survival. He would not be resuscitated should his heart stop. Flora described this as one of the hardest phone conversations she had ever had, not least because she was unable to be with Theo afterwards. The clinical staff on the study advisory panel clarified that visiting restrictions precluded a face‐to‐face family meeting in which these medical decisions could be explained, further highlighting the effects of the sudden rupture of familiar staff‐family relationships. Instead, clinicians had to resort to the phone to share bad news with family members, whom they had frequently never met in person.

Flora's account differed from others in our study in that the rupture of family involvement in ICUs was so sudden and prolonged. It is nevertheless similar in that it is markedly shaped by the chaos that marked the first weeks of the pandemic in the UK and the extreme difficulty families had trying to get updates from the hospital. For Flora, like for many others we spoke to, calling repeatedly was the only way to care from afar. And yet, Flora and others also worked hard *not to* call so as not to disturb busy staff, to allowing them to focus on providing care for ICU patients. This reflects the ambivalence that arose from the tension between trying to stay informed on the one hand and not being a nuisance to staff on the other.

Flora's account further highlights that despite the immense time and energy investments, the connections she was able to make were mostly fragile and temporary. When Theo's condition stabilized, his clinical team planned to move him to another hospital to free up his bed for sicker patients. While this made sense in organizational terms, for Flora it meant losing the carefully established links and rapport with staff. In other words, moving patients between hospitals frequently undid the building and rebuilding of relationships through which family members could be better informed and involved. Notably, Theo's move to the smaller hospital had a positive effect in the end: Flora received a direct number, could speak to the consultant there once a week, and could reach other staff more easily. The continuity in communication between family members and staff was thus partly dependent on the size of the hospital and on the ways in which family updates were organized. Looking back, Flora felt that there was too little communication with clinicians in the ICU, particularly when her husband—and, by extension, she—was most fragile. She emphasized that while she understood that this was an unusual and stressful time for staff, communication is a lifeline in a time when one cannot be present in the hospital. This remained traumatic for Flora, even months after Theo had come home in June 2020.

In part, experiences like Flora's reflect a tragedy of those very first admissions in March and April of 2020; concerted strategies improved communication as time went by. Patients we spoke to recounted that staff had encouraged them to call their families and were grateful for this, because they had themselves felt unable to do it either physically or emotionally (some had suffered from delusions and were convinced their families had either died or been estranged from them). Family members we spoke to generally appreciated the so‐called “family liaison” teams that hospitals later set up to reach out to those at home. The losses that ensued from the mandated physical separation, however, could never be entirely overcome: while phone calls were the best that could be offered at the time, even at a higher frequency, they fell short in fulfilling family members’ and patients’ need for physical co‐presence.

An ambivalence that persisted even as time progressed arose from the tension between informing others in the position of “the designated contact person” and looking after oneself. Many hospitals asked families to choose one person for them to be in touch with. Choosing who this should be was not straightforward for all families. Some chose the family member with the most “medical vocabulary.” Others felt it was self‐evident that this task should fall on them. Elizabeth, a former community nurse whose husband Jim died in the ICU in April 2020, described this as “a role she would want” but one she nevertheless found “extremely hard” and “absolutely exhausting.” In other words, while taking up this position allowed her to be involved in the care for her husband by being central to the communication about his ever‐changing condition, it also took an immense toll on her. In particular, she found it difficult to strike a balance between letting others hope for the best, and honesty about the critical nature of Jim's condition and the very real possibility of his death. In this position, family members took on a responsibility that otherwise usually fell to clinicians—that of accurately portraying medical information to various family members and friends and conveying bad news, but crucially without clinical training and experience.

From these abbreviated accounts we have seen how family members, patients, and staff sought to establish connections and positions that allowed them to care for one another when familiar modes of involvement were foreclosed due to visiting restrictions. For staff this included creative workarounds where systems and protocols no longer applied as before and were characteristically done while simultaneously attending to other obligations. The positions acquired through this choreographing of people and events were not straightforwardly easy or comfortable but instead were marked by ambivalence in that they were both wanted and burdensome. Much of this work took an emotional toll that weighed on them long after the hospital stay had ended.

## PERSONALIZING CARE AT A DISTANCE

Family members struggled to get a sense of what care in the ICU was like and how they might be involved from afar. As they could not take part in family meetings or share personal information about who their family member was “as a person,” and had in most cases not been approached to volunteer information about either medical history or social aspects (such as personal interests like music preference or hobbies) that could help shape the care in the ICU, they worried that the care for their relative would be impersonal, to the detriment of the patient. Our interviewees described this lack of knowledge as “anonymous” and equated the care given to “wartime medicine.”

In her desperation, Flora made “care packages” for the hospital staff. These contained things she and the children had baked; a card with the names of all the staff members Flora had spoken to on the phone; and to counter the sense of being “just a number,” added a photograph of the family. She also sought to support staff through this unprecedented, stressful time and to share with them stories and details about her husband to emphasize his being a person. These motivations, as we see it, were undoubtedly interconnected. We understand these as attempts to build relationships through which Flora and the children could personalize care for Theo at a distance.

Amina told a similar story. while her husband Omar was discharged shortly after his admission, her father Ahmed spent just under four weeks in hospital including two weeks in the ICU. Amina and her children made gift bags filled with creams and chocolates for staff and attached a card with a photo of Ahmed. In contrast to Flora, who was sometimes stopped from dropping things off out of the worry regarding contamination, Amina and her children could leave items with a nurse at the door to the ward after things had settled into more of a routine in December than they had in March 2020 and the virus had been established to be airborne. Amina also left coffee and sweets for Ahmed at the door of the ward. She found doing so comforting, as it “resembled normal life.” She managed to establish some degree of familiarity with staff via the phone and in‐person drop‐offs: on one occasion a nurse recognized Amina from previous drop‐offs and her daily calls.

The phone was again crucial to establishing connections and personalizing care. Amina tried to supplement the care her father was receiving via the phone, fearing that he would otherwise not receive the care he needed. Because she knew from friends and family about exercises that could bring up saturation levels, she talked him through these exercises on FaceTime and used the monitor to gauge the effects of his efforts. In doing so, she too took up a position otherwise taken up by clinical staff. Other interviewees recounted that nurses had reached out to update them about the care they had provided for the patient or to query what their hobbies and interests were.

We have seen that as a second way of crafting involvement in ICU care, family members and clinical staff sought to connect to exchange information about the patient, in some cases managing to establish tentative relationships through which physical distance could be overcome. It is such crafting of relationships that we turn to next.

## MAKING KIN AND VIRTUAL CO‐PRESENCE

Some family members could establish co‐presence in the ICU, either through videoconferencing technology or through staff they had known previously. The latter was true for Rose, whose husband Logan had been treated for non‐Hodgkin lymphoma a year before his admission to the ICU with COVID‐19 in April 2020. Rose spoke warmly about a videocall with a nurse who had asked Rose to stay on the line until she found a room where she could take her mask off. The small gesture of finding a room where she could take her mask off meant even more in a context in which time was extremely limited. The nurse remembered Rose from the time Logan had been in ICU a year earlier and said: “I've been following how well he's been doing, because I knew he was in, and I was so worried about him.” To Rose, having somebody in the ICU with Logan “who knew him before all of this happened” and had “met the kids” felt comforting, “almost like [having] a substitute member of the family.”

Similarly, ICU nurse Marian became a substitute family member to her close friend Byron, who was admitted to her unit when his family could not be present. When it became clear that Byron would not survive, Marian sang to him and stayed by his side during his final moments; her colleagues paused their work where they could as not to disturb them. While Marian appreciated her colleagues' respect, she worried that her grief added to their burden. This highlights the tension between caring for a dying friend and caring for colleagues in the ICU.

For end‐of‐life care, establishing virtual co‐presence was essential. For some family members, it was the only way to speak to and “see” their dying relatives, for in person visits were not always possible. This was the case for Nisha and Suri, whose father and husband, Ishan was admitted to a general hospital ward at the peak of the second wave, in January 2021. While his doctors were initially positive about Ishan's possibilities for recovery, things took a different turn: Ishan was admitted to the ICU on his tenth day in hospital, where he was mechanically ventilated. Before the clinical team intubated Ishan, Nisha and her father spoke via video call for although the hospital allowed in‐person end‐of‐life visits, both tested positive for COVID‐19 and could therefore not go to ICU. It would turn out to be the last time they spoke.

Nisha and Suri put their strength into staying hopeful for Ishan's survival. But when Ishan's kidneys failed a week later, he was placed on dialysis, and the doctors asked Suri and Nisha to come to hospital. Nisha still tested positive for COVID, and so she waited in the hospital car park when Suri went in and video‐called her and other family members from the ICU. They prayed for him. When Ishan developed a secondary infection and deteriorated despite high doses of steroids, Nisha and Suri were once again invited in to come in to say their goodbyes. This time, Nisha tested negative. She felt both “lucky,” in the sense that she still got to see him, and “scared” as her father's body had changed so much that he no longer looked like himself.

The next day, Suri and Nisha got a phone call from the doctor to invite them to join on video call as soon as possible; time was now limited. Once the connection had been established, Nisha and Suri spoke to Ishan, who was sedated and ventilated. They called Ishan's mother and other people close to him, hoping that hearing their voices would help him recover. Ishan's blood pressure continued to fluctuate and then dropped. Nisha recalled that the nurse stood next to her father, watching the monitor, and, putting her fingers on his pulse, said “I can feel a very faint pulse.” When she noticed that Nisha was fixated on the monitor, she moved it out of sight. Nisha continued: “Then she was holding his hand and stroking his head. That's something that I really appreciated. … If I was there or if my mum was there, that's what we would be doing. … That's what they did for us.” Then the nurse said she could no longer feel a pulse, and that Ishan was no longer alive. Suri and Nisha continued to speak to Ishan for another three hours, after which the nurse asked if they would like to talk to Ishan for longer, or receive a call back after they had taken the equipment off him, shaved his beard and “made him look presentable.” They opted for the latter option. They were also invited to the hospital morgue to see the body later that day.

Nisha's account highlights the significance of videocalls in choreographing distance and proximity between ICU patients and family at home. Although the connection established could never fully replace face‐to‐face contact, in which touch, to feel a pulse or to comfort, would have been possible, it could nevertheless help to enact proximity between family at home and those in the ICU.

## DISCUSSION

The stories we have told of disrupted family involvement in intensive care during the first months of the COVID‐19 pandemic in the UK highlight the isolation, fragmentation and loss that ensued from the (sudden) physical separation between patients and family members, and family members and staff. Many family members experienced this situation as traumatic, not only because of their relative's critical illness and proximity to death but also due to the radical disruption of routines and familiar modes of involvement in care. These findings echo those of other studies conducted in the UK and beyond that have highlighted the negative consequences of visiting restrictions for patients, family members, and staff, especially when someone died or was close to death (Block and Karb 2024; Digby et al., 2021; Hugelius et al., 2021; Robert et al., 2020).

At the same time, the stories we told also narrate desperate attempts to craft alternative modes of involvement through laboring to establish an embedded set of connections, personalizing care at a distance, and making kin and virtual co‐presence. Doing so enacted proximity despite physical distance. Where Ahlin reads frequent calling as a way of holding the fabric of family together and to provide care at a distance (Ahlin, 2020, 2023), we read the sending of “care packages” as a way to allow personalized information to travel from the home into the hospital and the building of relationships between staff and kin at home to be involved in care through them. But doing so could not always resolve the ambivalence that the physical separation had created. These findings resonate with existing work, detailing how people in novel situations develop innovative means for communicating and connecting with others, but doing so can feel stressful and laborious (Ennis‐Mcmillan & Costin, 2021). But in mobilizing various technologies for the crafting of proximity despite distance, responsibilities and relations were reordered (cf. Akrich, 1992; Oudshoorn, 2008)—effecting different and sometimes fraught experiences.

The only form of communication, initially, happened via telephone. For most family members, the burden of uncertainty, reaching out to the hospital (in the early days often with limited success) and waiting apprehensively by the phone, took its toll. Other work has also highlighted that the unpredictability of phone calls rendered family members unable to exercise any “control … over when and where they make themselves available” (Wajcman, 2015, p. 138), condemning them to an “always‐on” state of availability, and has called for the proactive scheduling of calls to ensure continuity of communication (Azoulay and Kentish‐Barnes, 2020; Robert et al., 2020). Family members could be better supported, for instance, through scheduling calls to family members to avoid their being “on call” constantly; supporting the delivery of bad news to other family members; and mental health support for staff members exposed to affective situations at work (see also Robert et al., 2020), although our analysis showed that such measures can inevitably only be partial solutions.

The introduction of videoconferencing could, and did, bring about some degree of proximity and togetherness; it presented families with a new way of “being with” the person in the hospital (Driessen et al., 2021). The infrastructures required for such connections took hospitals some time to establish, and it remained a high‐resource practice, particularly in terms of staff time, leading some hospitals to restrict access to it. They also took an emotional toll on staff, who often witnessed moments of intense grief as they were positioned within these encounters. Moreover, the existence of this medium of contact created expectations for staff to make it available, and communicating with family members that they could *not* use it (either due to staffing levels or guidance that stipulated terms of use) could induce additional stress. Lastly, the positive effects of technologically facilitated connections were not equitably available, as these strategies relied heavily on patient or families’ access to smartphones or computers, a stable internet connection, and technological literacy: those who did not have access to the technology or internet required, could not make use of it to be communicatively present and informed (however limited) in the same way.^8^

## CONCLUSION

In this article, we have sought to further nuance the conceptualization of care as reliant on physical proximity and of distance as necessarily “cold.” Facing a new disease with unpredictable outcomes, with limited knowledge about effective treatment options and no vaccine, NHS hospitals across the UK altered their visiting policies as part of the government's infection prevention measures. The visiting restrictions radically disrupted established modes of involvement of family members and patients. Drawing on John Law's conceptualization of care as a “choreography of separation” (2010) in which various “objects of care” and their accompanying subjectivities need to be cared for simultaneously, we have empirically attended to how proximity and distance reconfigured family involvement in ICU COVID‐19 care in the UK and novel subject positions for family and staff.

We showed how proximity could be enacted at a distance: through “care packages,” calls, and familiar faces. Our analysis thus highlights that care does not unproblematically flow from either proximity or separation but rather depends on their complicated orchestration. Our analysis also suggests that the ensuing subject positions are not fixed but instead dynamic and interconnected; that is, positions of staff or family do not exist independently of each other, as neither could provide the care expected of them in isolation, or indeed without the technologies that intermittently connected them.

We have drawn attention to the normative expectations and perceived responsibilities associated with each. Taking up the positions that afforded alternative modes of involvement in care did not come without a cost: to being a designated contact person was felt to be both desirable and burdensome, as it required balancing the preservation of hope with truthfulness about the possibility of death. When staff took on familial responsibilities, it was precisely their emotional involvement that could be distressing to colleagues. In other words, the ambivalence that emerged from the normative expectations associated with existing and emergent subject positions could not always be resolved. While we stop short of joining the call to query whether visiting restrictions are the most proportionate measure should another pandemic occur in the future (Hugelius et al., 2021; Jeitziner et al., 2022), we contend that, in the event of future pandemics, it is crucial to remain attentive to the effects of physical separation of patients and their close kin and to provide support where separation effects ambivalences, helplessness, anxiety, and isolation.

## ENDNOTES

1 See for a timeline of changing COVID‐19 policies in the UK: Institute for Government 2022, https://www.instituteforgovernment.org.uk/sites/default/files/2022‐12/timeline‐coronavirus‐lockdown‐december‐2021.pdf

2 Notably, the precise operationalization of these infection control measures differed between hospitals and changed over time. However, most allowed no visitors at all, with exceptions for end‐of‐life visits.

3 Visiting restrictions were just one of many infection control measures implemented in hospitals during the pandemic. It is therefore hard to ascribe a direct effect to them, although some studies have demonstrated a reduction of hospital transmission of respiratory viruses mor generally (not specifically SARS COv‐2) when compared with the absence of such restrictions, and a reduction in health care‐acquired respiratory viral infections (see Hugelius el al., 2021).

4 We understand family in the widest sense of the term, so that it is inclusive of blood relatives, partners, close friends, and other kin.

5 In a maximum variation sample one samples for research participants who are purposefully as different from each other as possible. Our key criteria for difference were age at the time of ICU admission, sex, (self‐reported) ethnicity, and time and duration of admission.

6 This module can be accessed via https://hexi.ox.ac.uk/Experiences‐of‐Covid‐19‐and‐Intensive‐Care

7 All participant names are pseudonyms.

8 Although we know of them, no families with this experience are part of our sample. For more on this limitation, see the methods section.
